# A Comparative Study of Chest CT With Lung Ultrasound After SARS-CoV-2 Infection in the Assessment of Pulmonary Lesions in Rhesus Monkeys (*Macaca Mulatta*)

**DOI:** 10.3389/fvets.2021.748635

**Published:** 2021-10-29

**Authors:** Chrispijn M. Schilp, Lisette Meijer, Martina Stocker, Jan A. M. Langermans, Jaco Bakker, Marieke A. Stammes

**Affiliations:** ^1^Biomedical Primate Research Centre (BPRC), Rijswijk, Netherlands; ^2^Department of Population Health Sciences, Unit Animals in Science and Society, Faculty of Veterinary Medicine, Utrecht University, Utrecht, Netherlands

**Keywords:** Ultrasound, computed tomography, SARS-CoV-2, COVID-19, non-human primate (NHP)

## Abstract

Lung ultrasound (LUS) is a fast and non-invasive modality for the diagnosis of several diseases. In humans, LUS is nowadays of additional value for bedside screening of hospitalized SARS-CoV-2 infected patients. However, the diagnostic value of LUS in SARS-CoV-2 infected rhesus monkeys, with mild-to-moderate disease, is unknown. The aim of this observational study was to explore correlations of the LUS appearance of abnormalities with COVID-19-related lesions detected on computed tomography (CT). There were 28 adult female rhesus monkeys infected with SARS-CoV-2 included in this study. Chest CT and LUS were obtained pre-infection and 2-, 7-, and 14-days post infection. Twenty-five animals were sub-genomic PCR positive in their nose/throat swab for at least 1 day. CT images were scored based on the degree of involvement for lung lobe. LUS was scored based on the aeration and abnormalities for each part of the lungs, blinded to CT findings. Most common lesions observed on CT were ground glass opacities (GGOs) and crazy paving patterns. With LUS, confluent or multiple B-lines with or without pleural abnormalities were observed which is corresponding with GGOs on CT. The agreement between the two modalities was similar over the examination days. Pleural line abnormalities were clearly observed with LUS, but could be easily missed on CT. Nevertheless, due to the air interface LUS was not able to examine the complete volume of the lung. The sensitivity of LUS was high though the diagnostic efficacy for mild-to-moderate disease, as seen in macaques, was relatively low. This leaves CT the imaging modality of choice for diagnosis, monitoring, and longitudinal assessment of a SARS-CoV-2 infection in macaques.

## Introduction

Ultrasonography, in general, is a highly sensitive imaging technique already widely used and nowadays also for bedside thoracic evaluation (1). Equipment is relatively cheap, easy to use, and can be deployed everywhere eliminating transport and radiation exposure in comparison to CT. For the diagnosis of lung pathologies with ultrasonography, ultrasound artifacts are used for interpretation. Air is the main component of healthy lungs which scatters and impedes the transmission of the sound waves. As the surface of the pleural wall is a strong reflector of the sound waves, it creates artifacts containing information about the lung pathophysiology (2). However, lesions that are not attached or associated with the pleural wall will be missed due to impedance of air in between the lesion and pleural wall. The viral agent severe acute respiratory syndrome coronavirus 2 (SARS-CoV-2) causes an infection in which the majority of the lesions seems to be located in the subpleural regions of the lungs, increasing the likelihood of appearance during lung ultrasound (LUS) and additional value of this modality (3–5).

SARS-CoV-2 that causes COVID-19 is responsible for an unprecedented global pandemic (6). Enormous scientific efforts in a broad range of fields have resulted in increased knowledge about this new disease, including the development of a wide range of vaccines as well as therapies. However, many aspects of disease development, including the long-term effects, are still unclear. Moreover, the development of new variants of the virus leading to faster spreading is of the utmost concern. To obtain more insights in the longitudinal progress of the disease process, experiments in controlled settings in an appropriate animal model are necessary. Non-human primates (NHPs), and more specifically rhesus monkeys, are such a model due to their close evolutionary relationship to humans, and because they show the mild-to-moderate symptoms seen in most human cases (7–9).

Experimentally SARS-CoV-2 infected rhesus monkeys are generally clinically asymptomatic, like the majority of the healthy human population, defining the need for reliable read-out parameters. Medical imaging, traditionally via chest X-rays (CXR) or computed tomography (CT) has already been widely used in both clinical and veterinary practice. Moreover, CT as an imaging modality to assess the longitudinal process of SARS-CoV-2 infection is already used in rhesus monkeys (9). SARS-CoV-2 infected rhesus monkeys often show pleural line irregularities and bilateral ground glass opacifications in the periphery of the lungs.

The aim of this study is to compare chest CT and LUS for the assessment of longitudinal SARS-CoV-2 infection in rhesus monkeys using a semi-quantitative scoring as an objective measure for pulmonary lesions. A strong correlation between the scores of both imaging techniques in different stages of infection could indicate that LUS could be an alternative for CT as a monitoring tool for COVID-19 disease patterns in both human and macaques.

## Materials and Methods

### Animals and Procedures

The study consisted of 28 Indian-origin, adult female rhesus monkeys (*Macaca mulatta*) (4–8 years of age, weighing 5.4–12.1 kg). The study was conducted at the Biomedical Primate Research Centre (BPRC, Rijswijk, Netherlands).

All macaques were purpose bred at the BPRC. During the study the animals were socially housed. Following SARS-CoV-2 infection the animal room was classified as animal biological safety level 3. All cages were provided with bedding and environmental enrichment. The macaques were offered a daily diet consisting of monkey pellets (Ssniff, Soest, Germany) supplemented with vegetables and fruit. Enrichment was provided daily in the form of fresh cut branches, mirrors, food puzzles, and a variety of other homemade or commercially available enrichment products. All procedures, husbandry, and housing performed in this study were in accordance with the Dutch laws on animal experimentation and the regulations for animal handling as described in EU Directive 63/2010. BPRC is accredited by the Association for Assessment and Accreditation of Laboratory Animal Care (AAALAC) International. The study was performed under a project license issued by the Competent Authorities (Central Committee for Animal Experiments, license no. AVD5020020209404). Before the start of the study, approval was obtained, by the institutional animal welfare body (CCD 028 Evaluation of vaccines and antiviral compounds against emerging coronavirus infections).

All procedures were performed in the morning and macaques were fasted overnight while water was available *ad libitum*. Macaques were anesthetized in their home cage with ketamine (10 mg/kg, ketamine hydrochloride, ketamine 10%; Alfasan Nederland BV, Woerden, Netherlands) combined with medetomidine hydrochloride (0.05 mg/kg, Sedastart; AST Farma B.V., Oudewater, Netherlands) intramuscular (i.m.) and subsequently transferred to the examination room. At the end of the procedure, when the macaques returned to their home cage, atipamezole (0.25 mg/kg, Sedastop; AST Farma B.V., Oudewater, Netherlands) was administered i.m. In addition, during every sedation bodyweight was measured.

### SARS-CoV-2 Infection

This study was performed as part of SARS-CoV-2 vaccine-related studies. No animals were infected solely for the purpose of this study. The macaques were infected with SARS-CoV-2 virus isolate BetaCoV/German/BavPat1/2020 p.4. The virus was obtained from the European Virus Archive (Charitéplatz 1, Berlin, Germany). The viral stock for the infection study was propagated on Vero E6 cells. Virus titer was determined using a 50% tissue culture infective dose (TCID_50_) assay on Vero E6 cells, as 1 × 10e5 TCID_50_/ml. The integrity of the virus stock was confirmed by sequence analysis. Virus infection was performed through the combined intranasal and intratracheal route. Virus was first diluted to 2 × 10^4^ TCID_50_ per mL. All macaques received 0.25 mL in each nostril and 4.5 mL intratracheally. Total dose was 1 × 10^5^ TCID_50_ (9). There were 25/28 animals that were sub-genomic PCR positive for at least 1 day in the throat sample.

### Computed Tomography (CT)

Non-invasive, free breathing, CT data were acquired pre-infection and 2-, 7-, and 14-days post-infection (p.i.) using a MultiScan Large Field of View Extreme Resolution Research Imager (LFER) 150 PET-CT (Mediso Medical Imaging Systems Ltd., Budapest, Hungary). The macaques were positioned headfirst supine with the arms up and fixated in a vacuum pillow. A single CT of the chest takes 35 s by which respiratory motion is inevitable. To mitigate the impact of respiratory motion and improve the image quality, respiratory gating was applied. The respiratory amplitude was detected with a gating pad placed next to the umbilicus. For the final reconstruction, the inspiration phases were automatically selected using MATLAB (10).

### Lung Ultrasound (LUS)

Lung ultrasound data was acquired directly pre- or post-CT to compare the outcome of both imaging techniques, on pre-infection, 2-, 7-, and 14-days p.i., using a Butterfly IQ, Point-of-Care Ultrasound device (Butterfly Network, Inc., Guilford, CT). Since the presets on this device were developed for human use, the preset “pediatric lung” was selected. The scanning scheme consisted of 10 zones covering the chest: one superior and inferior zone on the anterior and lateral sides and one inferior zone on the posterior side of each hemithorax (11, 12). The positioning of the macaques for the ventral zones was supine arms down, for the lateral zones supine arms abducted to a 90° angle and for the dorsal zones rotated on the contralateral side arms forward and up. The mid-clavicular-, axillar-, and scapular-line were used as reference to scan the lung in sagittal and coronal scanning planes, with a depth of ≤5 cm. Gain settings were adjusted to optimized visualization of the pleural line during the first scan of every individual macaque, the same gain setting were applied for all following timepoints.

### Data Analysis

#### CT Scoring

A semi-quantitative scoring system for chest CT was used to estimate SARS-CoV-2-induced lung disease (9, 13). Quantification of lesions on the CT was performed independently by two experienced imaging scientists based on the sum of the lobar scores. The degree of involvement in each zone was scored as: 0 for no involvement, 1 for <5%, 2 for 5–24%, 3 for 25–49%, 4 for 50–74%, and 5 for ≥75% involvement. An additional increase or decrease of 0.5 was used to indicate alterations in CT density of the lesions. A maximum score of 35 could be reached for the combined lobes per time point. An example of the representative CT scores observed in this study is visualized in Supplementary Figure 1. Pulmonary inflammation as a result of a SARS-CoV-2 infection often shows as a patchy and diffuse pattern of increased attenuation on CT. Most common is a hazy pattern, a so-called ground glass opacity (GGO) without infiltration in underlying vessels or bronchi. Another lesion type is a more localized pattern following thickening of the intralobular septa, a crazy paving pattern. The third lesion type found is less SARS-CoV-2 specific, a consolidation. A consolidation is an area of increased attenuation which can block underlying vessels or bronchi (14).

#### LUS Scoring

A semi-quantitative scoring system for LUS was used to estimate the degree of aeration (11, 15). Analysis of the LUS images was performed independently by a veterinarian and an imaging scientist. The degree of aeration in each zone was scored as: 0 for ≤2 B-lines, 1 for ≥3 B-lines or coalescent B-lines covering ≤50% of the screen, 2 for > 3 B-lines or for B-lines covering >50% of the screen, and 3 for lung consolidation. When subpleural alterations were scored, a “p” was added and an additional 0.5 was summed up to the score.

A maximum score of 30 could be reached for the combined zones per timepoint. The degree of involvement in LUS is a cumulative observation of artifacts caused by focal alterations of the pleural line (subpleural alterations) and within the subpleural tissue. Mild increase in density of subpleural lung tissue appears as a comet-tale from the pleural line to the bottom without fading (B-line). An increase in the number of comet-tales is recorded as multiple B-lines or, when showing a curtain-like pattern, coalescent B-lines. A consolidation is similar to what has been described for CT. An example of the representative LUS scores observed in this study is visualized in Supplementary Figure 2.

#### Standardizing

The scoring of CT differs from LUS since one includes a volume and the other a surface. To allow comparison of these two different scoring systems, the semi-quantitative scores were converted to qualitative scores. To achieve this, a score of 1 was given for all positive scores and a score of 0 was given for all negative scores.

To determine if the two methods agree qualitatively, the CT and LUS score of each separate region were summed up, leading to three possible outcomes; score 0 meaning that both modalities were negative, 1 meaning that one of the modalities was positive, and 2 that both modalities were positive. In this way comparison is relatively easy with score 0 and 2 as being the same, and 1 as being different.

**Figure 1:**
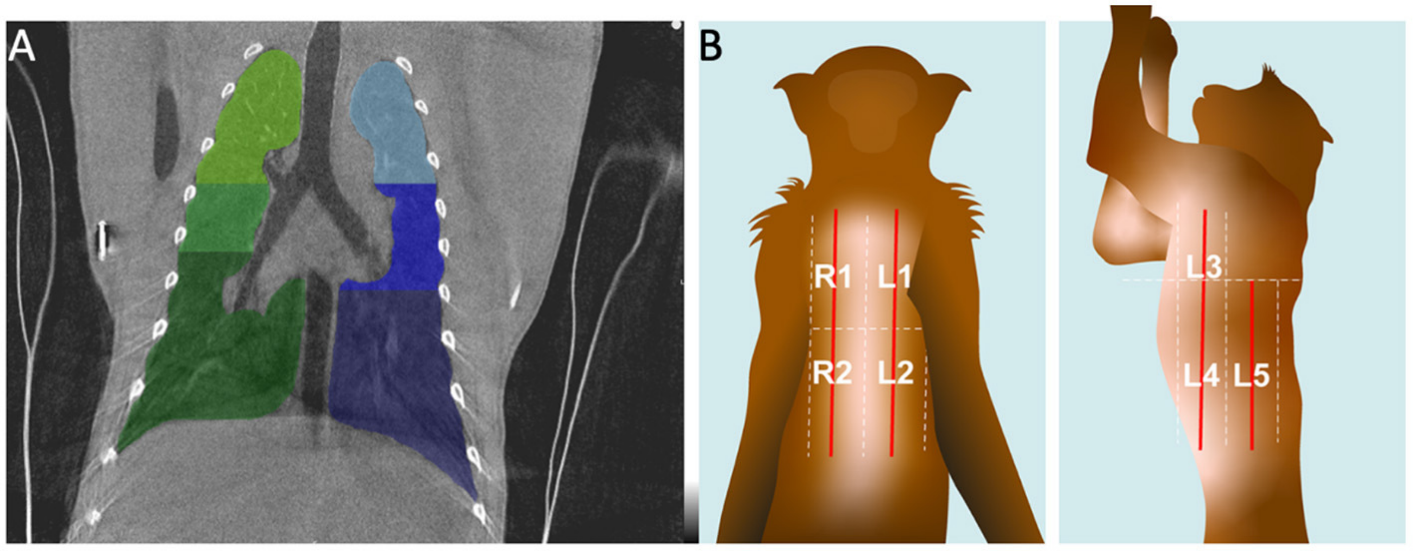
Visualization of the scoring zone of the lungs on CT **(A)** and LUS **(B)**. **(A)** A representative coronal CT image. The six zones are indicated with different colors. **(B)** At the graphic the arrangement of the five zones for LUS in dorsal and lateral recumbancy for the left side are illustrated.

Furthermore, the CT scoring system uses 6 scoring zones and LUS uses 10 scoring zones (Figure 1). For standardization, the lungs were divided in four areas: upper left, lower left, upper right, lower right. For CT the nipple level was used to divide the middle lobes. The upper and lower LUS scoring zones already had the border at nipple level, therefore we combined zones 1 and 3 and zones 2, 4, and 5 for left and right.

#### Statistical Analysis

To investigate whether the qualitative scores were representative for the semi-quantitative scores Spearman correlations were calculated. These correlations were conducted for each modality between the two scores (Prism, version 8.4.3).

In order to determine whether agreement (same/different) between the two modalities was influenced by the day the data was obtained (pre-infection, 2, 7, 14 p.i.) and, hence, the progression of the disease, a binomial model with a logit link was fitted with the function *glmer* of the package *lme4* (16) in R version 3.5.2 (17). This model included agreement (same/different) as response variable, day as predictor, and lastly, to control for repeated measures, subject as well as lung region nested within subject as random intercepts. As an overall test of the significance of the factor day, the fit of this full model was compared to a null model comprising the random intercepts only.

## Results

Chest CTs of the macaques revealed several manifestations of COVID-19 disease after SARS-CoV-2 infection. The macaques showed variability in time course and lung involvement. Most common lesions observed on CT were GGOs and crazy paving patterns. With LUS confluent or multiple B-lines with or without pleural abnormalities were observed which corresponds to GGOs on CT.

**Figure 2:**
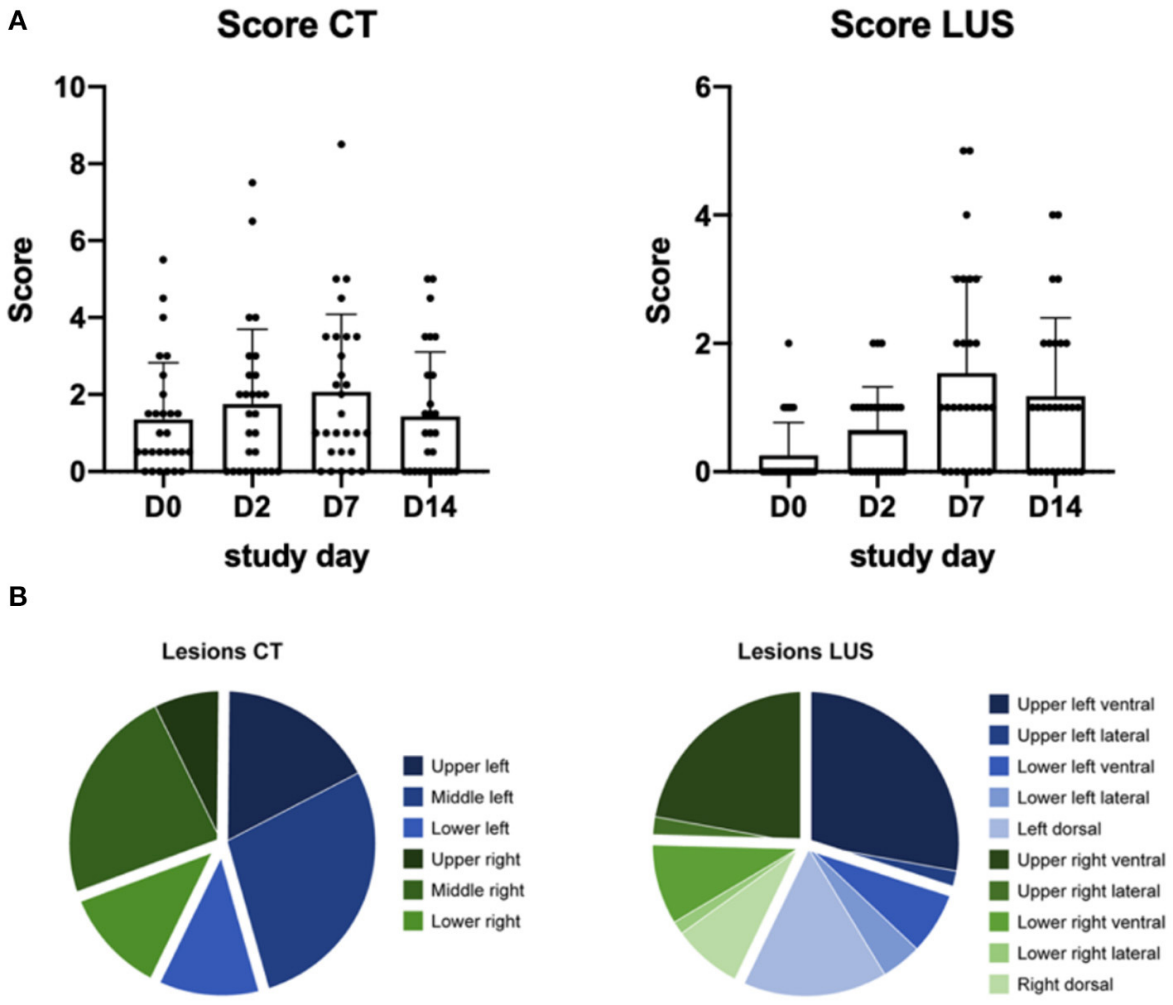
**(A)** Represents the semi-quantitative score of each study day, the dots represent each individual macaque. D0 is obtained pre-infection. Per day the average and standard error of mean are visualized. **(B)** The distribution of the lesions scored for each modality over all study days.

Semi-quantitative scores show a bell-shaped curve in the cumulative scores of both modalities (Figure 2A). Highest scores on CT (mean−2.0, range−0-8.5) and LUS (mean– 2.0, range−0-5.5) were scored at D7.

Distribution of all lesions showed that approximately 75% of the lesions observed on CT were in the upper and middle parts of the lung (Figure 2B). With LUS most lesions were detected in the upper regions and associated to the pleural line. The exact distribution of the lesions over the four planes and their respective correlation is visualized in Supplementary Figure 3.

**Figure 3:**
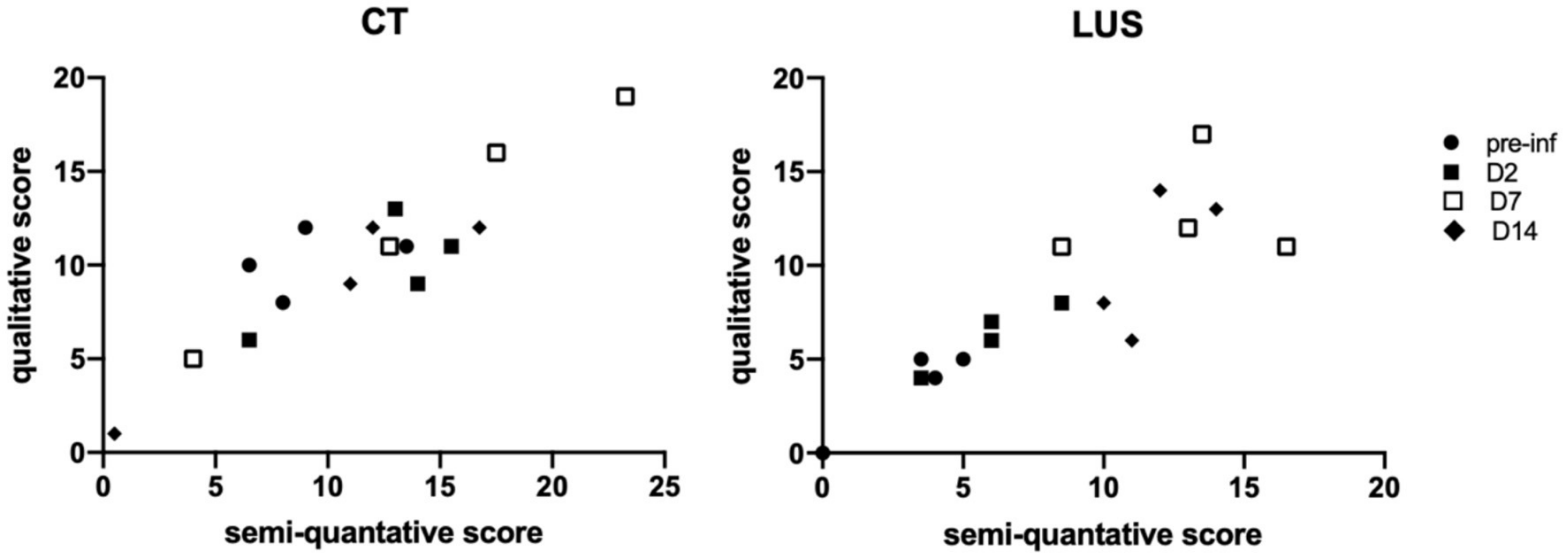
Correlation graphs between qualitative and semi-quantitative scores are shown per modality in which the days are marked with different symbols. The correlation for CT is r = 0.7778, p = 0.0006 and for LUS r = 0.8913, p < 0.0001.

For full comparison of both modalities a similar qualitative scoring system was applied, independent of the size of lesions. The correlation between the semi-quantitative scoring and the qualitative scoring for CT (r = 0.7778, *p* = 0.0006) and LUS (r = 0.8913, *p* < 0.0001) confirms that transforming the data is without substantial loss (Figure 3).

**Figure 4:**
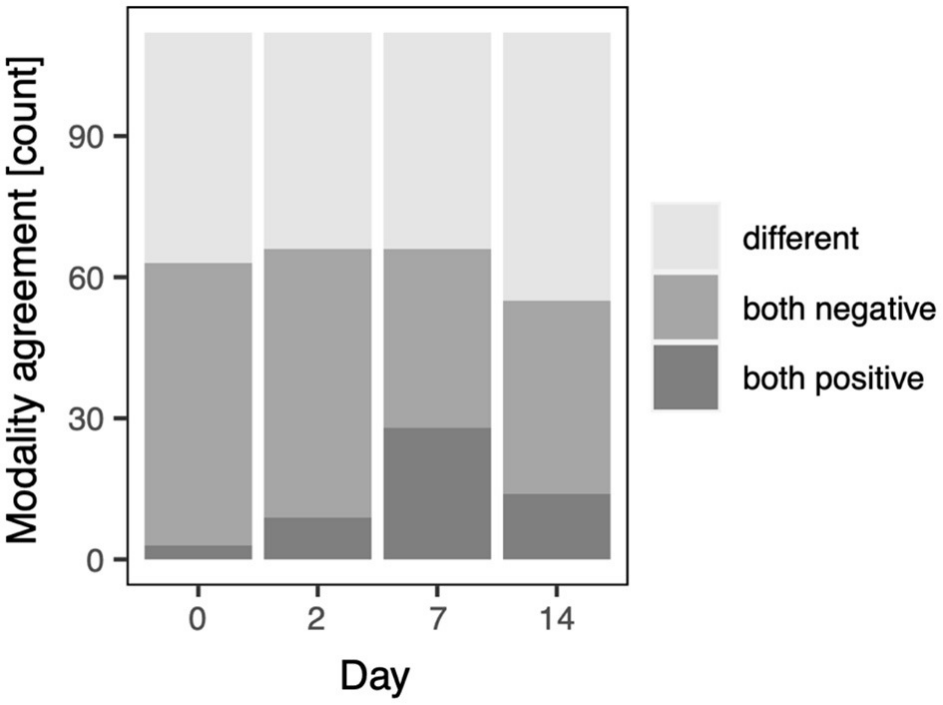
Modality agreement for the qualitative scores on the study days; pre-infection (D0), D2, D7, and D14, respectively.

With this qualitative data a binomial model was fitted to assess whether agreement between CT and LUS, i.e., same (both positive or negative) or different, is affected by the study day. The full-null model comparison revealed that the study day had no impact on the modality agreement (χ^2^ = 3.137, df = 3, *p* = 0.371). For the purpose of illustration (Figure 4) agreement between CT and LUS, modality agreement was split into both positive and both negative; note, however, that this was not included in the model investigating the impact of the study day.

**Figure 5:**
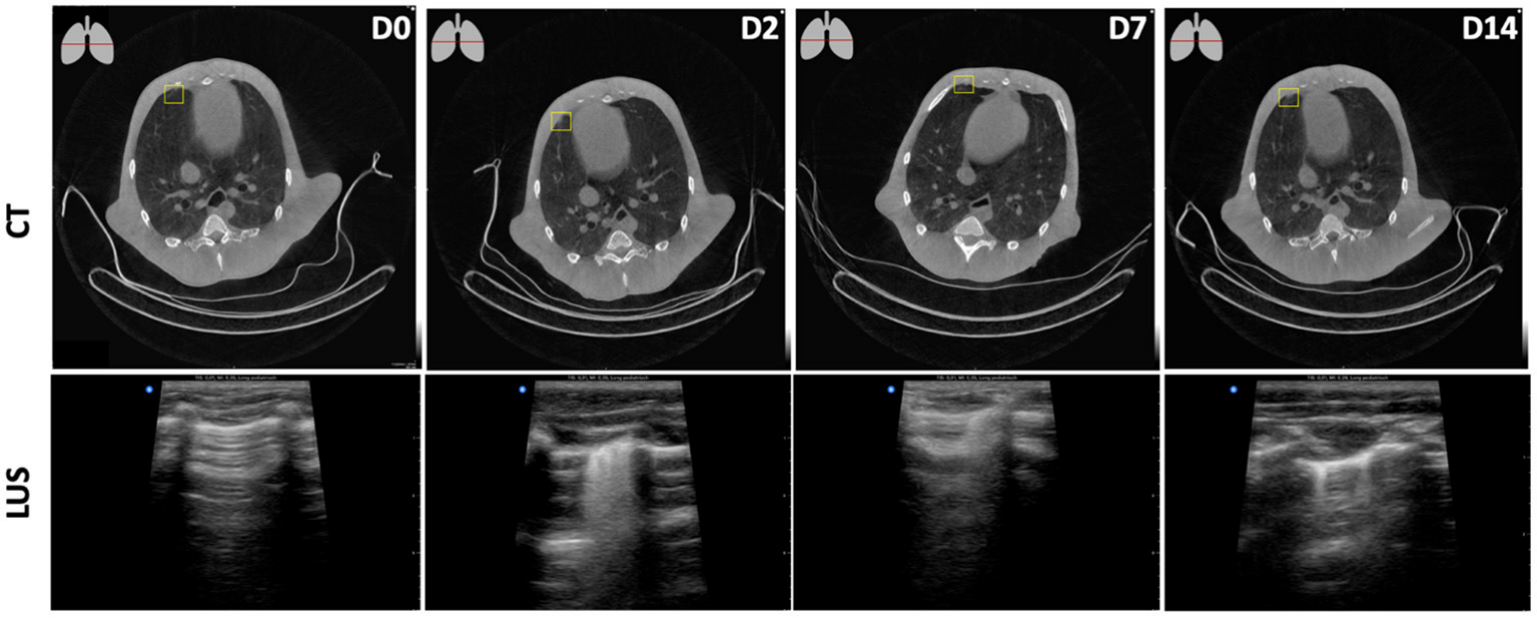
Longitudinal assessments of a peripheral focal nodular lesion after SARS-CoV-2 infection in the lower-right plane. D0, obtained pre-infection showed no aberrations both on CT and LUS. From D2 onwards, on CT (upper row) this minor consolidation was observed at the ventral side of the pleural wall in the right middle lobe. The lesion was detected in the lower right quadrant on the anterior pleural wall, corresponding with the R2 area on LUS (bottom row) leading to multifocal B-lines on D2, singular B-lines on D14, and a combination of both on D7.

The appearance of SARS-CoV-2 associated lesions on LUS and CT is visualized in Figure 5. In longitudinal assessments, a peripheral focal nodular lesion was observed in CT (upper row). The lesion was detected in the lower right quadrant on the anterior pleural wall, corresponding with R2 area on LUS (bottom row). While this could easily be missed on CT, LUS shows an irregular pleural line, resulting in a confluent pattern decreasing over time. On Day 14, this was degraded to a subpleural interruption and thickening of the pleura resulting in multifocal localized vertical artifacts, so-called B-lines. B-lines are heterogenous in their appearance which might be used to characterize the alterations in the lung parenchyma (15). The appearance of SARS-CoV-2 associated lesions on LUS in the other areas–upper-right, upper-left, and lower-left–are visualized in Supplementary Figure 4.

**Figure 6:**
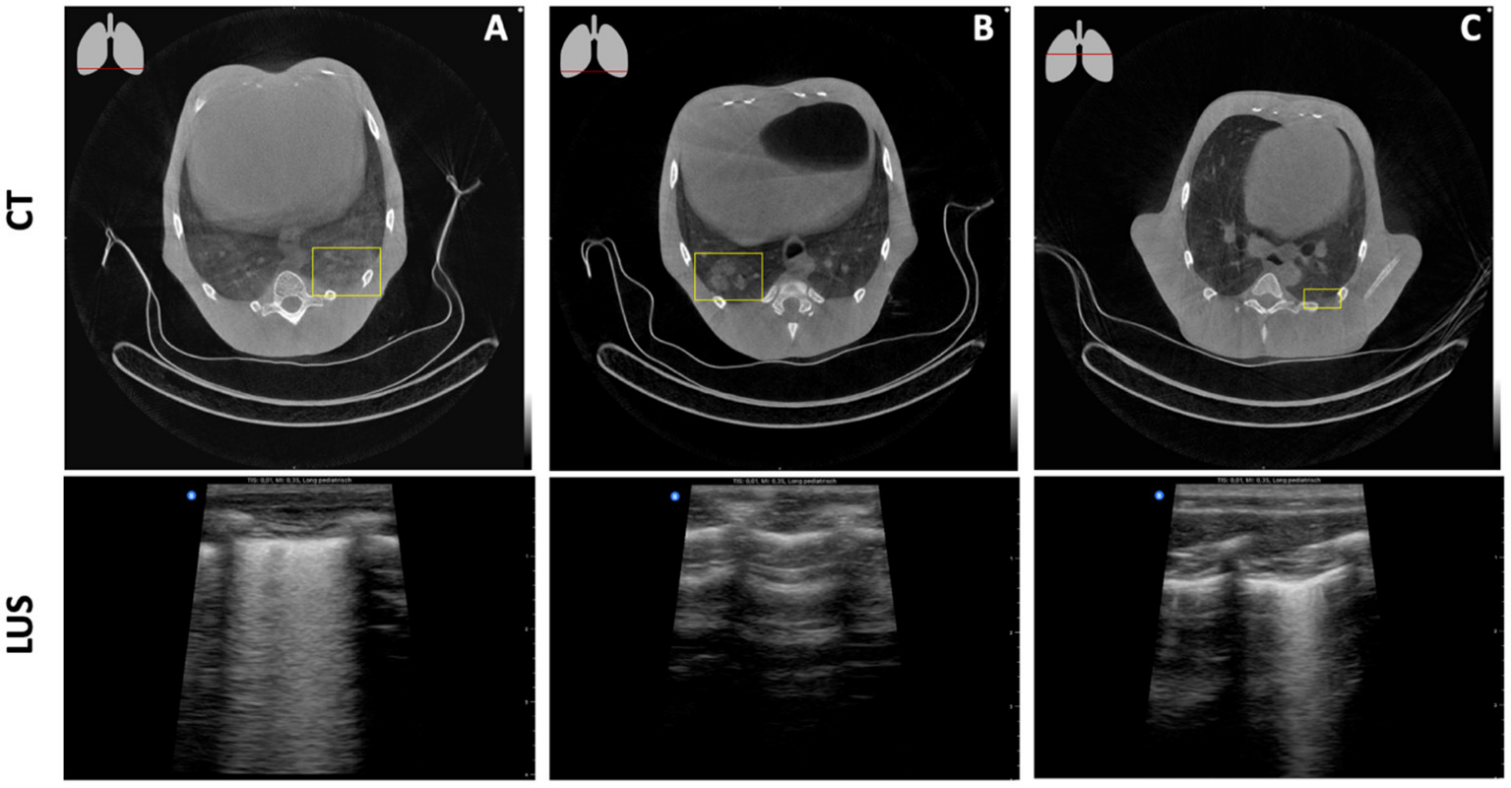
Detectability of lung lesions with LUS. **(A)** A ground glass opacity on CT, attached to the pleural wall, leads to a multifocal B-line pattern on LUS. **(B)** A consolidation on CT which is almost attached to the pleural wall results in a seemingly healthy lung with LUS. **(C)** A multifocal B-line pattern on LUS resolves a minor consolidation attached to the pleural wall on CT.

Lesions within lung parenchyma can only be detected when attached to the pleural wall with no aeration in between. When attached to the pleural wall they are clearly visible on LUS (Figure 6A). However, even the smallest amount of aeration absorbs the ultrasound signal, resulting in an image with seemingly healthy lung tissue (Figure 6B). Subpleural alterations can be very small and therefore challenging to see on CT. LUS, however, gives a clear image of pleural line abnormalities (Figure 6C).

## Discussion

In this study, we observed that with both chest CTs and LUS we can detect lung pathology after SARS-CoV-2 infection in rhesus monkeys and that the agreement between the two modalities is similar over the study days. The additional value of chest CTs in COVID-19 (18, 19) is already known but for LUS this is a new finding in macaques.

Ultrasound is a technique already extensively used in both clinical and veterinarian practice for a wide range of applications (3–5), though the use of ultrasound for visualizing the lungs is less common. This application is a development of the last years, which is highly interesting in the light of the current COVID-19 pandemic. It is interesting in this pandemic as a SARS-CoV-2 infection is predominantly observed in the respiratory tract and imaging is a tool for monitoring disease severity and for longitudinal follow-up of the lungs (4, 20, 21). In both adults and children, an improvement in the LUS score was found to be in accordance with the clinical conditions and laboratory tests (22). Though also the other way around is true, the number of lung areas with pathologic findings is positively associated with the need for ICU admission and/or mortality (23, 24). As in macaques, the disease severity is limited to a mild-to-moderate form with absence in almost all cases of clinical symptoms, it is challenging to find this same correlation between disease severity and LUS score, too. Nevertheless, the disease progression found in macaques mirrors that observed in humans without the progress associated with severe disease. Compared to human disease, CT abnormalities, predominantly GGOs, in macaques were detected in much earlier phases of disease with rapid resolution, albeit with considerable inter-subject heterogeneity; most lesions were first observed at 2 days p.i. and resolved within 2 weeks, with a peak around 7–10 days p.i. A similar overall pattern was observed for the viral load found in the swabs (nasal and tracheal) A similar overall pattern was observed for the viral load found in the swabs (nasal and tracheal) after a combined intratracheal and intranasal infection. However, the peak in viral load was already found on 2–4 days p.i. (9, 13, 25–28). This difference is likely due to the difference in location; with swabs the upper respiratory tract is evaluated, while with a chest CT the lower respiratory tract is visualized. In addition, no correlation has been detected between the viral load detected in swabs and disease severity (29), even though an increased CT score is an indication for worsening of the disease and can predict disease outcome (30, 31). Like what has been found in humans in our study no correlation could be detected between the viral load detected and imaging scores visualized (32). However, the lung abnormalities detected by CT were matched with gross pathology. For LUS, this matching was more challenging due to the description of the location of the abnormalities. In this study, only in one animal no lesions were observed on the CT on a study day by which 27/28 animals, with a maximum daily CT score of 8.5/35, have moderate disease following the guidelines on clinical severity of confirmed COVID-19 pneumonia (33).

In humans imaging is indicated for patients with a worsening respiratory status (34). In that regard, LUS can be considered as a primary monitoring tool for SARS-CoV-2 infection, with chest X-ray and CT as the reference standard (5, 34). This study confirms that longitudinal disease assessment is feasible and reliable as is also described for another acute respiratory disease severity model for NHP (15). Development of SARS-CoV-2 infection is initially associated in deeper bronchi characterized by particular thickening and irregularities of the pleural line followed by development of centralized lung lesions (35). In mild-to-moderate disease, these processes can develop independently of each other. The high sensitivity of LUS in the SARS-CoV-2 infection model is endorsed at both D7 and D14 when most of the pleural line abnormalities were observed with LUS, but could easily be missed on CT. Nevertheless, due to the air interface, ultrasound was not able to examine the complete volume of the lung. As a result, the severity of deeper parenchymal involvement during mild-to-moderate disease in NHP can easily be underestimated in contrast to a more severe human phenotype (4). Also, for humans the sensitivity is increased from 31.5% for a mild phenotype to 68.5% for a severe phenotype. Resulting in an overall sensitivity in humans of 60.3% when only scoring LUS patterns which are typical for a SARS-CoV-2 infection. While combining with less typical patterns the overall sensitivity is increased to 90.2% with sensitivities of 67.6% and 97.1% for the mild and severe phenotype, respectively (21). In this manuscript this last approach is applied to generate the highest sensitivity as possible.

LUS is mainly identifying artifacts, which is more subjective than observing lesions compared to CT. To generate a representative overview and score of the lungs, experience of the operator is required (36). In general, a competent operator is defined as someone with at least 25 patients scanned (37); this same amount can be applied to NHPs. In addition, the advantage of NHP scanning in a research setting over humans is that they are scanned immediately next to each other which increases the likelihood of higher reproducibility. Initially, the operator experience was limited with only 10 animals scanned. However, this possible confounding factor was resolved after including the 28 pre-infection scans. To quantify the alterations observed in the lungs with LUS, the method proposed by Soldati et al. (38) was used as a basis. However, due to the anatomical differences between humans and macaques and the flexibility of the shoulder region we decided to reduce the number of quadrants on the backside from three on each side to only the basal one. This reduced the total score which could be reached. This could be of impact for the comparison of the macaque data with human data. Nevertheless, as the level of disease severity found in macaques is only mild-to-moderate, maximum scores will not be reached and with this, comparison is still feasible.

The benefits associated with using LUS over CT in humans are only partly applicable to macaques in a research setting. The reduction of radiation exposure, following the as low as reasonably acceptable (ALARA) guidelines, is always good. However, the benefit for the bedside approach by which no transport is needed and reduction of the risk of exposure to SARS-CoV-2 in other rooms or to other persons is absent as the animals still need to be sedated and will be transported to a different room for examination (22, 24).

Further research could include different NHP species to confirm the applicability of LUS among species. In addition, it could also include other animal species like dogs and cats in which LUS has proven to be a useful technique but also warrants further investigation to use as a stand-alone imaging modality (39, 40). Moreover, to assess the full potential of LUS in NHPs, the effect of different levels of disease severity with respect to the diagnostic efficacy of LUS over CT needs to be evaluated probably in other lung disease models like tuberculosis or influenza (41, 42).

In conclusion, like what has been observed in humans, the sensitivity of LUS is high though the diagnostic efficacy for mild-to-moderate disease is relatively low (4, 43). This low efficacy outweighs the advantages of LUS over CT, i.e., no need to transport the animals and the absence of ionizing radiation. For this reason, CT is still the imaging modality of choice for diagnosis, monitoring, and longitudinal assessment of a SARS-CoV-2 infection in NHPs.

## Data Availability Statement

The original contributions presented in the study are included in the article/Supplementary Material, further inquiries can be directed to the corresponding authors.

## Ethics Statement

The study was performed under a project license issued by the Competent Authorities (Central Committee for Animal Experiments, license no. AVD5020020209404). Before the start of the study, approval was obtained, by the institutional animal welfare body (CCD 028 Evaluation of vaccines and antiviral compounds against emerging coronavirus infections).

## Author Contributions

CS, LM, and MSta: conceptualization, investigation, and writing—original draft preparation. CS, LM, MSto, JB, and MSta: methodology. MSto: investigation. CS, LM, MSto, and MSta: formal analysis. JL and MSta: supervision. All authors writing—review and editing, read, and agreed to the published version of the manuscript.

## Conflict of Interest

The authors declare that the research was conducted in the absence of any commercial or financial relationships that could be construed as a potential conflict of interest.

## Publisher's Note

All claims expressed in this article are solely those of the authors and do not necessarily represent those of their affiliated organizations, or those of the publisher, the editors and the reviewers. Any product that may be evaluated in this article, or claim that may be made by its manufacturer, is not guaranteed or endorsed by the publisher.
