# Supplementary material for: A Comparative Study of Chest CT With Lung Ultrasound After SARS-CoV-2 Infection in the Assessment of Pulmonary Lesions in Rhesus Monkeys (Macaca Mulatta)
Source: Front Vet Sci. 2021 Oct 29;8:748635. doi: 10.3389/fvets.2021.748635 (PMC8585853; doi:10.3389/fvets.2021.748635)
Supplement: Supplementary file 1 [file Data_Sheet_1.pdf]

Supplementary material

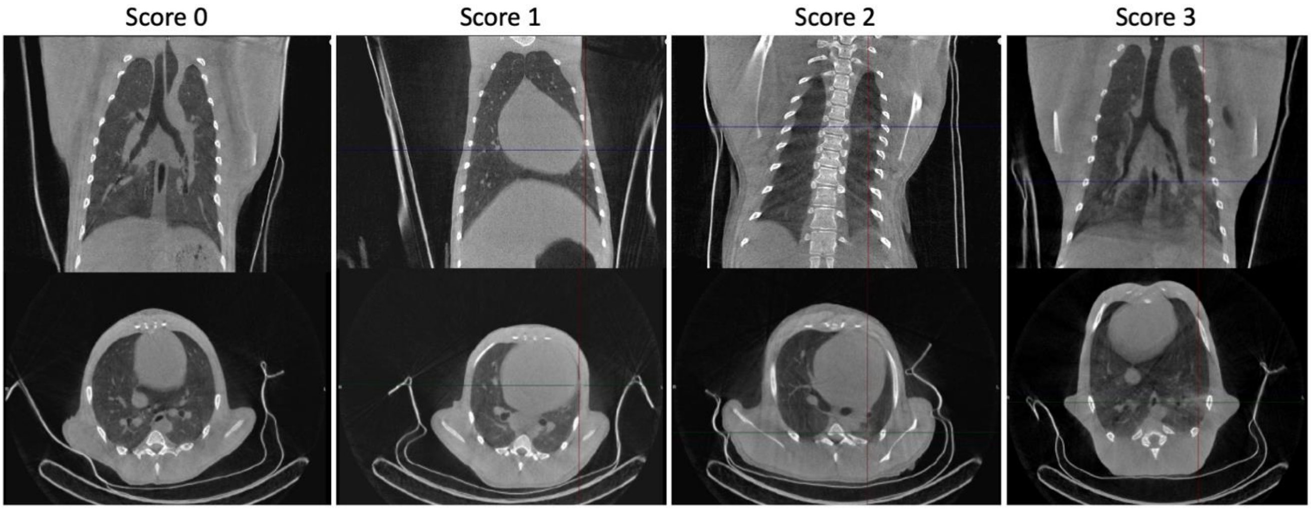

Supplementary Figure 1*: Visualization of representative CT scores based on the degree of involvement per lung lobe.*

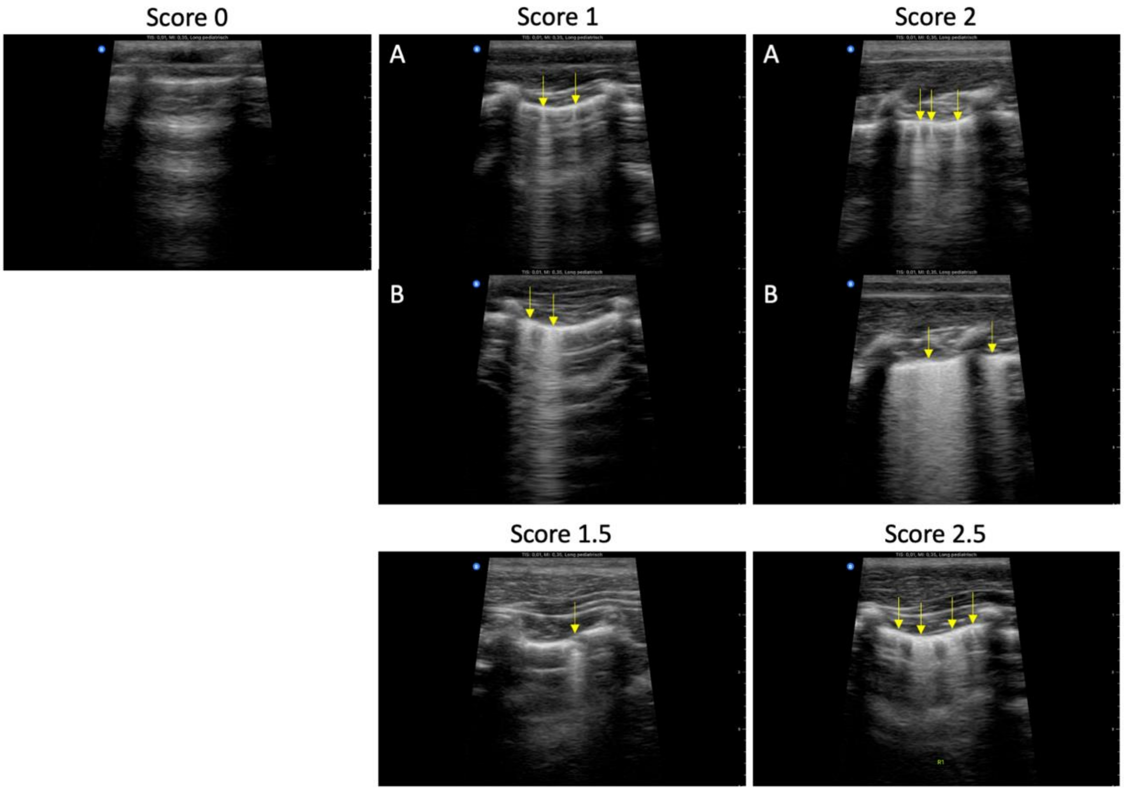

Supplementary Figure 2*: Visualization of representative LUS scores based on the degree of aeration. When subpleural alterations were scored, an additional 0.5 was summed up to the score. The* ***A-pictures*** *show an example of score 1 and 2 involving separate B-lines; The* ***B-pictures*** *show an example of scores 1 and 2 involving coalescent B-lines.*

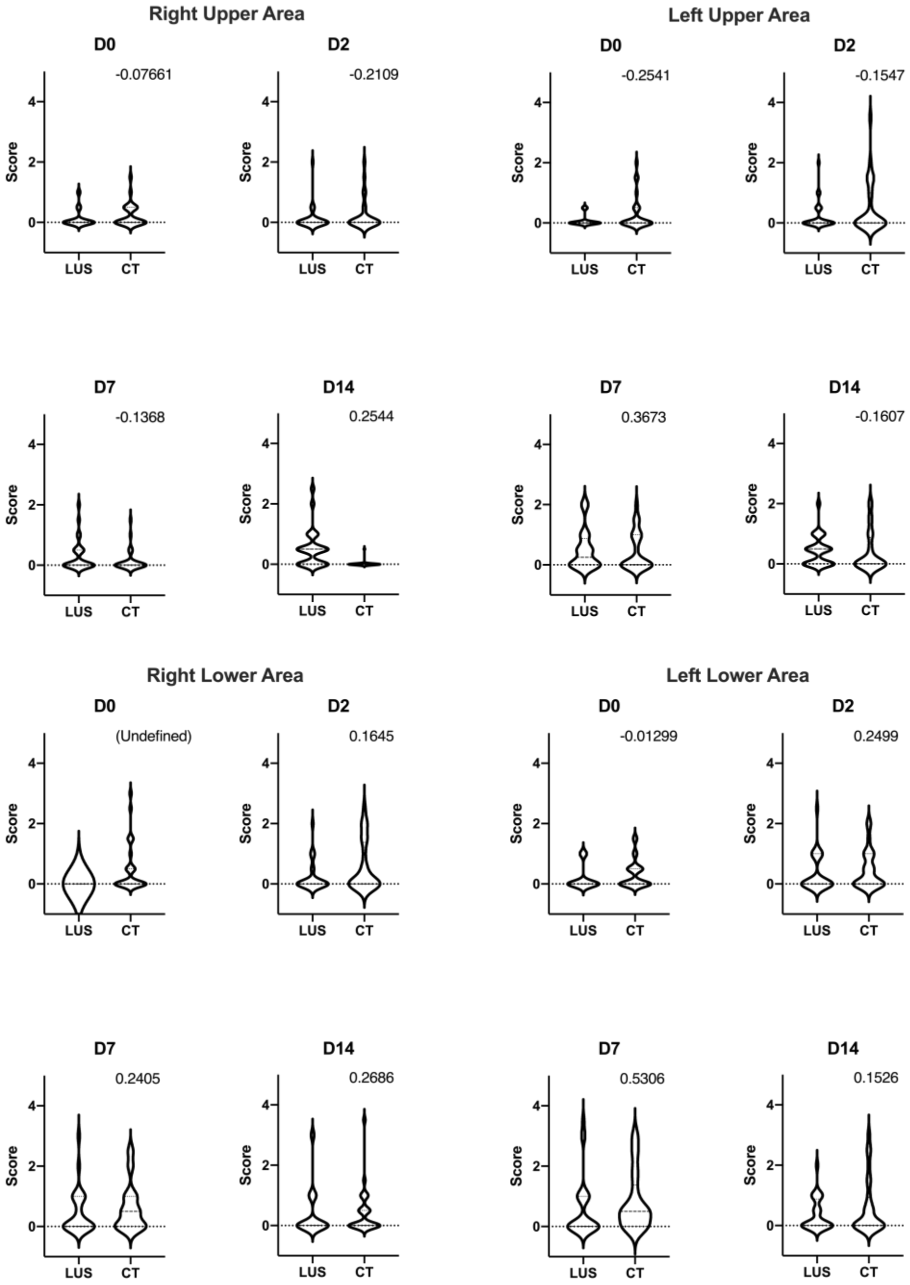

Supplementary Figure 3*: Graphs showing the daily scores of the separate areas on both LUS and CT. The Spearman correlation is calculated for each area and timepoint, the corresponding r-values are indicated in the upper right corner of each graph. Right-upper area (R1 and R3), right-lower (R2, R4 and R5), left-upper (L1 and L3), left-lower (L2, L4 and L5).*

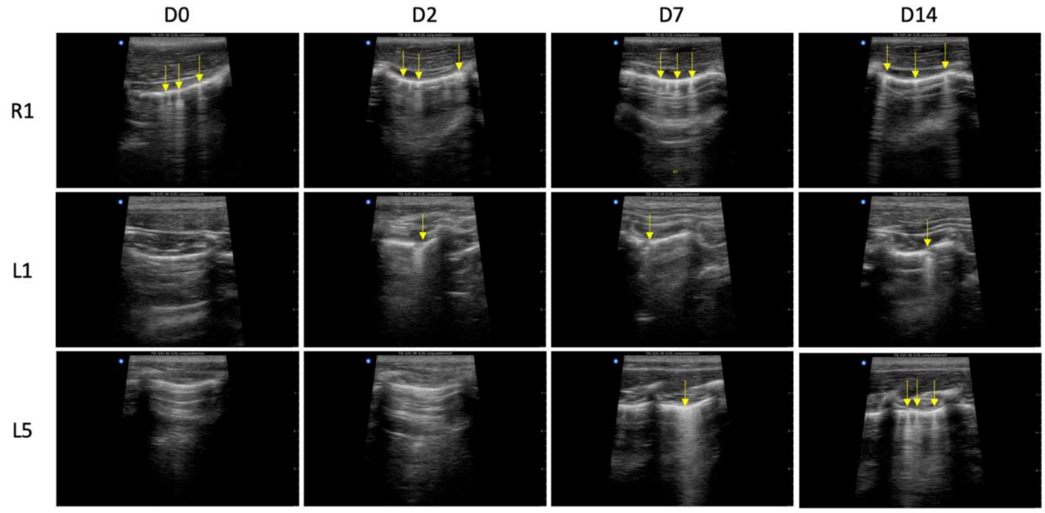

Supplementary Figure 4*: LUS images showing the lesion development, within a specific region, over time. The upper row (R1) shows an example of multiple B-lines; the middle row (L1) of a subpleural alteration. The bottom row (L5) presents an example of coalescent B-lines resulting in a curtain-like pattern (D7) and resolving in multiple B-lines on D14. The irregularities are marked with yellow arrows.*
